# Rapid containment of nosocomial transmission of a rare community-acquired methicillin-resistant *Staphylococcus aureus* (CA-MRSA) clone, responsible for the Staphylococcal Scalded Skin Syndrome (SSSS)

**DOI:** 10.1186/s13052-016-0323-y

**Published:** 2017-01-06

**Authors:** Onofrio Lamanna, Dafne Bongiorno, Lisa Bertoncello, Stefano Grandesso, Sandra Mazzucato, Giovanni Battista Pozzan, Mario Cutrone, Michela Chirico, Flavia Baesso, Pierluigi Brugnaro, Viviana Cafiso, Stefania Stefani, Floriana Campanile

**Affiliations:** 1 Ospedale dell’Angelo, Mestre, Venice Italy; 2 MMARLab - Department of Biomedical and Biotechnological Sciences (BIOMETEC), University of Catania, Via Santa Sofia 97, 95123 Catania, Italy

**Keywords:** Cross-transmission, ST5/*spa* type t311 CA-MRSA clone, ET_A_ exfoliative-toxin

## Abstract

**Background:**

The aims of this study were to identify the source and the transmission pathway for a Staphylococcal Scalded Skin Syndrome (SSSS) outbreak in a maternity setting in Italy over 2 months, during 2014; to implement appropriate control measures in order to prevent the epidemic spread within the maternity ward; and to identify the Methicillin-Resistant *Staphylococcus aureus* (MRSA) epidemic clone.

**Methods:**

Epidemiological and microbiological investigations, based on phenotyping and genotyping methods, were performed. All neonates involved in the outbreak underwent clinical and microbiological investigations to detect the cause of illness. Parents and healthcare workers were screened for *Staphylococcus aureus* to identify asymptomatic carriers.

**Results:**

The SSSS outbreak was due to the cross-transmission of a rare clone of ST5-CA-MRSA-SCC*mec*V-*spa* type t311, exfoliative toxin A-producer, isolated from three neonates, one mother (from her nose and from dermatological lesions due to pre-existing hand eczema) and from a nurse (colonized in her nose by this microorganism). The epidemiological and microbiological investigation confirmed these as two potential carriers.

**Conclusions:**

A rapid containment of these infections was obtained only after implementation of robust swabbing of mothers and healthcare workers. The use of molecular methodologies for typing was able to identify all carriers and to trace the transmission.

## Background

Staphylococcal Scalded Skin Syndrome (SSSS) describes a spectrum of superficial blistering skin disorders caused by the exfoliative (or epidermolytic) toxins of *Staphylococcus aureus*, predominantly, but not exclusively, affecting neonates and children under the age of 5 years [1].

Clinically, SSSS in children usually starts within 24–48 h with fever, irritability and widespread redness of the skin, and fluid-filled blisters form that rupture easily, leaving an area that looks like a burn. Typical characteristics of the rash include: tissue-paper-like wrinkling of the skin, followed by the appearance of large fluid-filled blisters in the armpits, groin and body orifices such as the nose and ears; the rash quickly spreads to other parts of the body including the arms, legs and trunk. In newborns, lesions are often found in the diaper area, axillary folds and around the navel; the top layer of skin begins peeling off in sheets, leaving a moist, red and tender area exposed. Other symptoms may include weakness and dehydration [2–4].

In humans, SSSS is caused by the release of two exfoliative epidermolytic toxins, ET_A_ or ET_B_, from *Staphylococcus aureus* toxigenic strains. These toxins induce epidermal blistering through the cleavage of the cell-cell adhesion molecule desmoglein-1, which is only expressed by keratinocytes in the granular cell layer, leading to a spectrum of illnesses ranging from mild localized blistering to extensive generalized lesions [2, 5]. ETs producing *S. aureus* strains have been associated with specific genetic backgrounds, on the basis of their phage types, accessory gene regulator (*agr*) groups and macrorestriction profiles [3–10].

SSSS in newborns has been mainly reported in clonal complexes (CC) 121 and 15, carrying *eta* and/or *etb* genes [2], but also in other less common genotypes, such as ET_B_-positive ST91 MRSA and ET_A_-positive ST2993-t211 (CC8) with and without SCC*mec*V [11, 12].

The prognosis in most cases is typically good if prompt antibiotic therapy is given. Approximately 5% of *S. aureus* strains produce exfoliative toxins, with a variable prevalence index in different countries. In Europe, the incidence of SSSS varies from 0.56 cases/million/year in France, to 2.53 cases/million/year in the Czech Republic [2, 3].

Recently, a nosocomial outbreak of SSSS has been described in neonates in England sustained by *S. aureus* isolates belonging to *spa*-type t346 [2].

Our study reports the experience of an SSSS outbreak, due to the cross transmission of a methicillin-resistant *S.aureus* isolate belonging to a rare clone of ST5-CA-MRSA-SCC*mec*V-*spa* type t311, ET_A_ toxin producer, among three infants hospitalized in the maternity ward of an Italian hospital. Rapid containment of these infections was obtained only after implementation of robust swabbing of healthcare workers, and the use of molecular methodologies for typing that was able to identify carriers and to trace the transmission.

The aims of this study were: i) to identify the causative agent and the transmission pathway; ii) to enforce correct control measures avoiding the spread within the maternity ward; iii) to characterize the MRSA epidemic clone.

## Methods

### Setting

The outbreak occurred in the Maternity Unit of the hospital “Dell’Angelo” in Mestre (Venice), Italy, with 40 beds, about 1,800 delivers per year, 7 delivery rooms, 2 operating rooms and one nursery, where the neonates stay when they are not in the room with their mother. The Maternity Unit has 112 Health Care Workers (HCW).

### Clinical investigations

During the cluster period, from November 20 to December 4, 2014, from the date of the first birth to the date of the discharge of the third newborn, 66 neonates were born in the unit. Umbilical swabs and other skin lesion swabs were performed on neonates presenting with clinical scalded skin syndrome signs. During the hospital admission, the neonates affected by SSSS were cared for in single rooms with their parents and swabs and digital photographs of skin lesions were taken.

### Parent and staff screening investigations

Parents and relatives of the ill new-borns were screened by nasal swabs, and hand swabs only in cases of skin lesions.

The maternity staff screening for *S. aureus* carriers started on December 6 by nasal swabs. Visual hand inspection and anamnesis research for hand skin diseases were conducted among the operators involved in the sick neonates’ pathway from the delivery room to the nursery and mothers’ room. All staff voluntarily complied with the request for screening.

### Infection control measures

During the progress of the investigation, the following control measures were applied: (i) immediate repetition to all the operators about contact measures for the prevention of infectious diseases and recommendation to the nursery operators to use a hand wash with a hydro-alcoholic solution before handling each neonate; (ii) immediate use of barrier measures (masks and gloves) for all the operators, until the identification of the index case. Operators usually used gloves only during delivery; (iii) immediate onset of screening to find *S. aureus* carriers; (iv) on December 9, extraordinary environmental sanitation in delivery rooms, nursery and ward rooms. In the nursery, in addition to the classic cleaning procedures, on December 18, a hydrogen peroxide and silver cation nebulization machine was used; (v) from December 12, topic mupirocin treatment was applied; (vi) on December 23 and January 8, two meetings with doctors, nurses, midwifes and support staff were held for clinical audit about the procedures followed from delivery to the neonates hospital discharge and discussion about the strategy used.

### Microbiological investigations

Nose and hand swabs were collected from doctors, nurses, midwives and support staff involved in the sick newborns’ management, from the delivery room to the nursery, as well as from the neonates’ parents and from the skin lesions of the sick newborns. All samples were cultured on Columbia Blood Agar (CBA) and Mannitol Salt Agar (MSA) plates (Becton Dickinson GmbH, Heidelberg, Germany); colony identification was obtained using coagulase test and Vitek2 GP card (BioMerieux, Marcy-L’Etoile, France) and Aris Sensititre ITGPOSF was used for susceptibility tests (Trek Diagnostics, Cleveland, OH, USA).

All the culture plates positive for *S. aureus* were sent to the “Medical Molecular Microbiology and Antibiotic Resistance” reference laboratory (M.M.A.R.L. http://www.labmicrobiologia.unict.it), Department of Biomedical and Biotechnological Science (BIOMETEC), University of Catania, for further characterization.

Methicillin resistance was re-evaluated by the cefoxitin disk diffusion method and correlated with the presence of the *mec*A gene, as suggested by the European Committee on Antimicrobial Susceptibility Testing (EUCAST) and Clinical and Laboratory Standards Institute (CLSI) guidelines [13, 14]. The antimicrobial susceptibilities were evaluated in agreement with the EUCAST interpretation criteria [13]. The *S.aureus* isolates were also screened by population analysis (PAP/AUC) to verify the presence of vancomycin-intermediate (VISA) and heteroresistant vancomycin-intermediate S*. aureus* (hVISA) phenotypes [15].

Molecular typing, *agr* locus and the virulence gene content (adhesion and toxin genes) were performed as previously described [16–20]. Representative *S. aureus* control strains were included in the study for phenotypic and genotypic assays [16, 17].

## Results

### Clinical presentation of the outbreak and case series

The outbreak of SSSS infections occurred in neonates from November to December 2014 in the Maternity Unit of the hospital “Dell’Angelo” in Mestre (Venice), Italy.

Three cases were identified over a 2-month period. Patients were born from 20 to 25 November 2014. All hospitalization periods overlapped, suggesting possible cross-transmission from one neonate to another.

In all cases, the skin symptoms began after 3–9 days from birth. After birth, they were discharged but returned to the hospital in 2–5 days. After four days from the admission of the third neonate, the Medical Chief of the pediatrics department reported this to the hospital Director of the Infectious Control Team (ICT) who then activated a Task Force for the epidemiological and microbiological investigation. All patient skin swabs were positive for MRSA. Blood cultures remained negative. None of the neonates required intensive care after delivery and none had an invasive procedure.

### Screening investigations

During hospitalization, several control measures were implemented to prevent epidemic spread and an attempt was made to identify the source of pathogen transmission.

Multiple samples were collected from neonate mothers and from the hospital staff.

Among the mothers, case 1 mother had dermatological lesions due to pre-existing hand eczema. On December 12, she was found positive for MRSA strains by the rapid Vitek identification method, both in her nose and on her hands. Case 2 mother was not colonized by *S.aureus*. Case 3 mother was found positive for *S.aureus* by nasal swab.

Twenty-seven HCW (24.1%) were positives for *S. aureus*, 3 (2.7%) of them for MRSA. On December 12, the treatment with nasal mupirocin was started (twice a day, for 5 days) only for those operators that were MRSA positive, and those that were waiting for the microbiological response from the laboratory. Only for the carriers of the same cluster strain, was a new control swab taken after 5 days from the end of therapy. They were successfully decolonized. No further neonate or staff-member has been identified with MRSA strains to date; the last controls were made on 25 March, 2015.

### Microbiological characteristics

In the course of the neonatal, parent and staff screening investigations, 9 *S.aureus* strains from affected skin regions, nasal swabs and carriers were isolated and sent to the reference lab (MMARL) of the BIOMETEC Department of the University of Catania, for further characterizations.

The microbiological and molecular characteristics of the sample in study are shown in Table 1.

**Table 1:** Clinical and microbiological characteristics of MRSA/MSSA isolates from SSSS and contact screening

Code	Date of isolate	Case type	Clinical feature	Case definition	Speciment	PFGE type	agr type	ST-SCC*mec-spa* type	Toxin genes detected				
									*luk*E	*luk*S/F	*eta*	*ica*A	*fnb*A
s1	06/12/2014	staff	NC	Colonized	Nares	C1	II	5-V-t311	+	-	+	+	+
s2	09/12/2014	staff	NC	Colonized	Nares	A	II	5-IV-t1094	+	+	+	+	+
s3	10/12/2014	staff	NC	Colonized	Nares	B	I	8-II-t3240	+	+	+	+	+
s4	11/12/2014	staff	NC	Colonized	Nares	D	III	*mec*A negative^b^	+	-	-	+	+
n 1	02/12/2014	neonate	SSSS	Infected	Affected skin (jaw, gluteus)	C1	II	5-V-t311	+	-	+	+	+
n 2	29/11/2014	neonate	SSSS	Infected	Affected skin-groin	C2	II	5-V-t311	+	-	+	+	+
n 3	04/12/2014	neonate	SSSS	Infected	Affected skin - leg	C2	II	5-V-t311	+	-	+	+	+
m 1	06/12/2014	mother	^a^SSTI	Infected ?	Nares, hand lesions	C2	II	5-V-t311	+	-	+	+	+
m 3	09/12/2014	mother	NC	Colonized	Nares	E	I	*mec*A negative^b^	-	-	-	+	+

*NC* no clinical signs, *SSSS* staphylococcal scaled skin sindrome, *agr* accessory gene regulator locus, *ST* sequence type, *SCCmec* staphylococcal chromosomal cassette

^a^SSTI: dermatological lesion (hand eczema); ^b^MLST and spa-typing were restricted only to methicillin-resistant isolates

Seven out of the 9 swab cultures yielded methicillin-resistant *S.aureus*, only resistant to beta-lactams and susceptible to levofloxacin, gentamicin, erythromycin, clindamycin, trimethoprim-sulfamethoxazole, tetracycline and rifampicin. The MIC values for the main anti-Gram positive drugs (linezolid, vancomycin, teicoplanin and daptomycin) were in the range of full susceptibility. Population analysis confirmed that all strains were vancomycin-susceptible, without glycopeptides heteroresistant sub-populations.

Two methicillin-susceptible *S.aureus* strains (MSSA), *mec*A gene negative and cefoxitin susceptible, and susceptible to all antimicrobials tested, were found in case 3 mother and in a staff-member.

All strains isolated from the three neonates, the case 1 mother (index-case) and one nurse showed a typical CA-MRSA phenotype and belonged to the same clone ST5-SCC*mec*V-*spa* type t311, with the same PFGE profile C (subtypes C1 and C2) and *agr*-locus II. The virulence gene content revealed that all isolates were positive for the *eta* gene, encoding for the epidermolytic toxin responsible for SSSS, and positive for *luk*E, *ica*A, *fnb*A, *sej* genes. No other toxin genes were present in these cases, including *luk*S-*luk*F (Panton-Valentine leukocidin), *tst, sea, seb, sec, sek/q* genes and the ACME-locus.

Two staff members were found to carry two non-outbreak MRSA strains, belonging to ST5-SCC*mec* IV-t1094 *agr*II (formerly pediatric or USA800 HA-MRSA clone) and ST8-SCC*mec* II-t3240 *agr*I (formerly Irish clone). They had different PFGE-types (A and B), and possessed the same toxin gene content with the addition of the Panton-Valentine Leukocidin gene.

## Discussion

Our study describes an outbreak of SSSS in a maternity setting in Italy over 2 months during 2014.

Surveillance of the staff members by nasal swabs identified *S.aureus* carriage in 24.1% of HCW. This percentage is close to those reported by other authors from Ireland (30%) and England (21%) [2, 3, 21–23]. MRSA strains were found on three nurses (2.7%), of which only one showed the characteristics of the outbreak strain. It was possible to eradicate *S. aureus* and the nurse was still decolonized after 4 months. After an accurate analysis of the work shifts, it was possible to establish that the operator was always on duty during the care of the three ill newborns in the nursery.

The molecular characterization highlighted the spread of a rare CA-MRSA clone ST5-*spa* type t311, unusually associated with the SCC*mec*V cassette of veterinary origin. This association has rarely been described in the literature [24]. This clone (already known as USA800 or pediatric clone) is usually associated with the SCC*mec* IV element, and it is widely spread in both hospitals and the community [25]. From these data, it can be assumed that there was a possible horizontal transfer of the SCC*mec* V cassette in a different genetic background, commonly diffused in the hospital setting.

In this study, ST5-SCC*mec*V-*spa*-type t311 epidemic strains producing ET_A_ toxin, isolated from the three neonates, case 1 mother and the nurse were indistinguishable.

Retrospectively, based on the microbiological and molecular data, two different routes can explain this neonate infection. The mother of one of the newborns was affected by chronic eczema, and was found to carry the epidemic strain; she might have been an asymptomatic carrier on admission to the unit, and she might have infected her child. Nasal, axillar and perianal carriage of *S.aureus* strains producing epidermolytic toxin have been reported in 3% of pregnant women [26, 27]. Moreover, atopic chronic dermatitis (also known as atopic eczema) has been recognized as an important source of skin infection, responsible for the dissemination of *S.aureus* in hospitals [28].

The other possibility is that the nurse was the source of the epidemic strain and might have infected the three neonates, one of which might have infected its own mother. In hospitals and in nurseries, outbreaks of staphylococcal infections are expected to originate from asymptomatically colonized care attendants rather than mothers [29, 30].

### Conclusions

The validity of this survey was to promptly find the causative agent of SSSS, to prevent the spread of the pathogen within the hospital ward, and to identify the MRSA epidemic clone. In fact, the available mitigation measures, such as screening of parents and hospital staff that was immediately implemented, stopped the epidemic in less than a week after onset.

The immediate implementation of strong barrier measures was enough to break the spread of the infection. The nurse, decolonized after some months, is still employed in the same department.
